# Plasma urate concentrations and possible REM sleep behavior disorder

**DOI:** 10.1002/acn3.50929

**Published:** 2019-11-12

**Authors:** Yun Shen, Junjuan Li, Michael Schwarzschild, Milena Pavlova, Songbin He, Alberto Ascherio, Shouling Wu, Liufu Cui, Xiang Gao

**Affiliations:** ^1^ Department of Neurology The Second Affiliated Hospital of Soochow University Suzhou China; ^2^ Department of Nephrology Kailuan General Hospital Tangshan China; ^3^ Department of Neurology Massachusetts General Hospital Harvard Medical School Boston Massachusetts; ^4^ Department of Neurology Brigham and Women’s Hospital Harvard Medical School Boston Massachusetts; ^5^ Department of Neurology Zhoushan Hospital Wenzhou Medical University Zhoushan China; ^6^ Department of Nutrition and Epidemiology Harvard School of Public Health Boston Massachusetts; ^7^ Department of Cardiology Kailuan General Hospital Tangshan China; ^8^ Department of Rheumatology Kailuan General Hospital Tangshan China; ^9^ Department of Nutritional Sciences The Pennsylvania State University University Park Pennsylvania

## Abstract

**Objective:**

To examine how urate concentrations are related to the risk of having possible REM sleep behavior disorder (pRBD) in a community‐based cohort.

**Methods:**

The study included 12,923 Chinese adults of the Kailuan Study, free of Parkinson disease (PD) and dementia. Plasma urate concentrations were measured in 2006, 2008, and 2010. Cumulative average urate concentration was used as primary exposure. In 2012, we determined pRBD status using a validated RBD questionnaire‐Hong Kong (RBDQ‐HK). Logistic regression analysis was performed to estimate the association between urate concentrations during 2006–2010 and odds of having pRBD in 2012 or pRBD case with symptom onset within 1 year.

**Results:**

Higher average urate concentrations were associated with a lower odds of pRBD (*P*‐trend <0.001). The adjusted odds ratio (OR), for the highest versus lowest urate quintiles, was 0.43 (95% confidence intervals (CIs) 0.32–0.57). Significant association was consistently observed when we examined the association of a single urate assessment (2006 or 2010) or the rate of change in urate concentrations during 2006–2010 with pRBD (*P*‐trend <0.001 for all). However, restricting to pRBD onset during 2011–2012, we observed a nonsignificant trend between high urate concentration and high odds of pRBD (*P*‐trend = 0.09).

**Interpretation:**

Higher average urate concentrations were associated with a lower likelihood of having pRBD, but not new‐onset pRBD. Because of its observational study design, the result should be interpreted with caution due to the possibility of residual confounding.

## Introduction

REM sleep behavior disorder (RBD), characterized by abnormal sleep behaviors of dream enactment, has been increasingly recognized as a highly specific marker of *α*‐synucleinopathies, and considered a non‐motor manifestation and the strongest marker of prodromal of Parkinson disease (PD).^1^, ^2^ RBD precedes clinically overt neurodegenerative disorders, with 73.5% converting after 12‐year follow‐up.^3^

Recent studies have consistently shown that higher concentrations of urate, a potent natural antioxidant with neuroprotective properties,^4^, ^5^ are associated with a lower risk of developing PD and with a slower rate of PD progression.^6^, ^7^, ^8^ However, it remains unknown whether urate status is associated with altered odds of having RBD. This is of significance in clinical and public health practices because it could improve our understanding of the potential role of urate in the pathogenesis of PD and help to develop a primordial prevention strategy against PD risk.

We thus conducted a community‐based study to examine the association between urate concentrations, which were repeatedly assessed in 2006, 2008, and 2010, and subsequent likelihood of having possible RBD (pRBD) in 2012 among ~12,000 adults. We hypothesize that higher plasma urate concentrations are associated with lower risk of having RBD.

## Methods

### Participants

The current study was based on an ongoing Chinese cohort, the Kailuan Study (The Chinese Clinical Trial Registry #: ChiCTR‐TNRC‐1101489).^9^, ^10^, ^11^, ^12^, ^13^ As detailed elsewhere, the current pRBD study included 12,990 participants (10,725 men and 2265 women) in the Kailuan Study, free of neurodegenerative disorders.^9^, ^10^, ^11^ We further excluded 67 participants who did not have urate assessment, leaving 12,923 participants (10,680 men and 2243 women) in the current analyses.

### Standard protocol approvals, registrations, and patient consents

The study was approved jointly by the Ethics Committee of the Kailuan General Hospital and the Human Subjects Committee at Brigham and Women's Hospital/Harvard Medical School.

### Assessment of pRBD

We used a validated RBD questionnaire‐Hong Kong (RBDQ‐HK) to determine pRBD status in 2012. The RBDQ‐HK, which is a self‐administered questionnaire that can be filled in also by or in collaboration with the bed partner, comprises 13 questions assessing different clinical features of RBD.^14^ The total RBDQ‐HK score is calculated by summing up the scores of all lifetime items and last‐year frequency items, giving a range from 0 to 100 and the cutoff point to estimate RBD for the total scale was >18.^14^ Our previous validation study conducted in Chinese adults suggested that the RBDQ‐HK has moderate sensitivity (84.4%), specificity (81.0%), and positive predictive value (70.8%), high internal consistency, and test–retest reliability.^15^ Similar results were reported in another validation study (e.g., 86.3% positive predictive value).^14^

### Assessment of plasma urate concentration

Study participants returned fasting blood samples in the morning after an 8‐ to 12‐h overnight fast in the 2006, 2008, and 2010 surveys. Samples were transfused into vacuum tubes containing EDTA (Ethylenediaminetetraacetic acid), collected and measured at the central laboratory of the Kailuan General Hospital. We determined concentrations of urate, using an autoanalyzer (Hitachi 747; Hitachi, Tokyo, Japan), as described elsewhere.^16^ The coefficient of variations of serum urate assessment was ≤6.0% for both within and between groups.

We calculated cumulative average of repeated measurements of serum urate during 2006–2010 to represent long‐term urate level. We calculated the changes between adjacent measurements and calculated per year change variable to represent changes in serum urate from 2006 to 2010.

### Measurement of covariates

Information on education level, income level, marital status, occupation, physical activity, smoking status, drinking status, tea consumption, past medical history of myocardial infarction (MI), stroke, cancer history, hypertension, diabetes, and head injury was collected in 2006 and updated every 2 years via questionnaires, as detailed elsewhere.^9^, ^11^ Weight, height, and blood pressure were assessed by trained field workers (i.e., physicians and nurses). Body mass index (BMI) was calculated for each participant based on his or her weight and height (weight/height^2^). Hypertension was defined as systolic blood pressure ≥ 140 mm Hg or diastolic blood pressure ≥ 90 mmHg or the use of antihypertensive medications in the last 2 weeks regardless of blood pressure status. It was the average of the two readings for analysis.

We assessed concentrations of triglyceride, low‐density lipoprotein cholesterol (LDL‐c), and high‐density lipoprotein cholesterol (HDL‐c) using an autoanalyzer (Hitachi 747; Hitachi, Tokyo, Japan) at the central laboratory of the Kailuan General Hospital.

Information on several sleep parameters including insomnia, daytime sleepiness, obstructive sleep apnea (OSA), and use of hypnotics was collected in 2012 and 2014. Insomnia status was assessed via a validated Chinese version of the Athens Insomnia Scale (AIS),^17^ which consists of eight measurement items of sleep features, with the cutoff score of 6, based on the balance between the sensitivity (93%) and specificity (85%). Daytime sleepiness was assessed via a validated Chinese version of Epworth Sleepiness Scale (ESS).^18^ A total ESS score ≥10 was considered as excessive daytime sleepiness. OSA was assessed with the snoring, tiredness, observed apnea and high blood pressure‐BMI, age, neck circumference, gender (STOP‐BANG) questionnaire and neck circumference, which was measured by trained field workers in 2014.^19^ Previous validation studies showed the high performance of the STOP‐BANG questionnaire with the sensitivity (91–94%) among different populations, including Chinese.^20^ In this study, participants with intermediate or high risk of OSA (STOP‐BANG score ≥ 3) were considered having OSA.^19^

### Statistical analyses

All statistical analyses were conducted using SAS version 9.4 (SAS Institute, Inc, Cary, NC). Continuous urate concentrations were divided into categories based on quintile. Cumulative average urate concentrations during 2006–2010 were used as primary exposure. A single assessment of urate in 2006 (the earliest available urate data) and in 2010 (the latest urate value before pRBD assessment), and rate of change in urate during 2006–2010 were used as secondary exposure.

We used logistic regression to calculate odds ratio (OR) values and 95% confidence intervals (CIs), and to test the odds of having pRBD across the different quintiles of urate. Trends in OR for pRBD risk across urate concentrations quintiles were determined considering urate concentrations quintile as an ordinal variable. Potential confounders were adjusted, including age, sex, educational level (primary, middle, college), income level (<600 RMB/month, 600–1000 RMB/month, >1000 RMB/month), marital status (single, married, divorced), occupation(white collar, blue collar, coalminer), physical activity( never, <4 times/week, every time more than 20 min; ≥4 times/week, every time more than 20 min), smoking status(never, past smoker, current smoker), drinking status(never, past drinker, current drinker), tea consumption (never; <4 times/week; ≥4 times/week), MI history (no, yes), stroke history (no, yes), cancer history (no, yes), hypertension (no, prehypertension, hypertension), diabetes (no, prediabetes, diabetes), BMI (<24 kg/m^2^, 24–28 kg/m^2^, ≥28 kg/m^2^), triglyceride(<0.82 mmol/L, 0.82–1.22 mmol/L, 1.22–1.87 mmol/L, ≥1.87 mmol/L), LDL (<1.76 mmol/L, 1.76–2.12 mmol/L, 2.12–2.65 mmol/L, ≥2.65 mmol/L), and HDL (<1.31 mmol/L, 1.31–1.54 mmol/L, 1.54–1.79 mmol/L, ≥1.79 mmol/L).

Because a sex difference in the urate–PD relationship was observed in previous study,^7^ we examined whether the potential association between urate and pRBD was modified by sex, by including multiplicative terms in the logistic models, with adjustment for aforementioned covariates. We further explored interactions between urate and age, BMI (<24, 24–28, or ≥28 kg/m^2^), and hypertension (yes/no).

To minimize the potential effect of RBD mimics on the observed association between urate and pRBD, we conducted several sensitivity analyses by excluding participants with other sleep disorders (e.g., OSA, insomnia, daytime sleepiness, or use of hypnotics), and alcohol drinkers. Because chronic diseases, such as cancer and cardiovascular disease, and related treatment, could have impact on both urate status and sleep pattern, we conducted a sensitivity analysis by excluding participants who had these conditions before and during the follow‐up. We conducted another sensitivity analysis using the alternative cutoff point (range 0–70, >7) for determining pRBD based on seven subgroup behavioral factors of RBDQ‐HK, as detailed elsewhere.^10^, ^11^ To understand the potential temporal relation between urate and pRBD status, we also conducted a sensitivity analysis by restricting to the pRBD case whose symptom appeared in the past year (i.e., symptom onset in 2011–2012).

## Results

The average age was 54.0 years in 2006. The demographic characteristics, health behaviors, and clinical diagnoses in the different level of urate concentrations were summarized in Table S1. Participants with high urate concentrations were more likely to be men, coalminers by occupation, current smokers, alcohol drinkers, tea drinkers, and at a higher level of education and income, and to have a higher prevalence of MI history, stroke history, and higher BMI, and concentrations of triglyceride and LDL‐c.

In 2012, a total of 727 participants (98 women and 629 men) were determined as pRBD. Higher average urate concentrations were associated with a lower risk of having pRBD (*P*‐trend <0.001). After multivariable adjustment, participants in the top quintile of urate had lower odds for pRBD (adjusted OR = 0.43; 95% CIs 0.32–0.57), compared with the bottom quintile of urate. The adjusted OR of each 100 *μ*mol/L increase in urate was 0.61(95% CIs 0.54–0.70). We observed similar significant results when we used a single urate assessment (2006 or 2010) to predict pRBD (adjusted OR comparing two extreme quintiles was 0.51 for the 2006 urate and 0.33 for the 2010 urate; *P*‐trend <0.001 for both). Significant association was consistently observed using the rate of change in urate concentrations during 2006–2010 as exposures (adjusted OR 0.53 comparing two extreme quintiles, 95% CIs 0.41–0.68; adjusted OR 0.87 of each 100 *μ*mol/L increase in urate, 95% CIs 0.82–0.91) to predict odds of having pRBD. Results did not change materially in the sensitivity analyses using alternate pRBD definition and excluding participants with RBD mimics, such as other sleep disorders, or those who reported alcohol consumption during 2006–2012. Similar results were obtained after we exclude those with major chronic diseases in or prior to 2012. We observed a positive trend between higher urate concentration and higher odds of new‐onset pRBD. (Table 1).

**Table 1:** The odds ratios (ORs) and 95% confidence intervals (95%CIs) of having probable RBD, according to urate status, among 12,923 adults

	Quintile of urate concentrations						*P*‐trend
	Q1	Q2	Q3	Q4	Q5	OR for each 100 *μ*mol/L increase in uric acid	
Average Uric acid between 2006–2010, range	<243 *μ*mol/L	243–291 *μ*mol/L	291–335 *μ*mol/L	335–388 *μ*mol/L	>=388 *μ*mol/L		
Case/N	205/2519	205/2882	123/2726	106/2632	88/2164	‐	‐
Sex‐age‐adjust	1 ref	0.83 (0.67–1.01)	0.48 (0.38–0.61)	0.42 (0.33–0.54)	0.42 (0.32–0.55)	0.61 (0.54–0.68)	<0.001
Full–adjust^1^	1 ref	0.84 (0.68–1.03)	0.50 (0.40–0.64)	0.43 (0.33–0.55)	0.43 (0.32–0.57)	0.61 (0.54–0.70)	<0.001
Using the alternative definition of pRBD^2^	1 ref	0.87 (0.71–1.06)	0.53 (0.42–0.67)	0.44 (0.34–0.56)	0.49 (0.38–0.64)	0.65 (0.58–0.73)	<0.001
Restricting to 8147 participants without insomnia, OSA, and did not use hypnotics^1^	1 ref	0.90 (0.68–1.19)	0.43 (0.31–0.61)	0.34 (0.24–0.50)	0.36 (0.24–0.53)	0.54 (0.50–0.64)	<0.001
Restricting to 10,045 participants free of MI, stroke, and cancer in and prior to 2012^1^	1 ref	0.82 (0.64–1.03)	0.46 (0.35–0.61)	0.37 (0.27–0.50)	0.38 (0.27–0. 53)	0.57 (0.49–0.66)	<0.001
Restricting to 3,361 participants who did not any drink alcoholic beverages in or prior to 2012^1^	1 ref	0.81 (0.54–1.21)	0.68 (0.41–1.10)	0.39 (0.20–0.76)	0.68 (0.33–1.40)	0.69 (0.51–0.94)	0.02
Using pRBD with symptom onset in the past year^3^	1 ref	1.05 (0.73–1.52)	1.17 (0.81–1.70)	1.06 (0.71–1.57)	1.17 (0.77–1.78)	1.07 (0.89–1.28)	0.09
Urate concentration in 2006	<235 *μ*mol/L	235–279 *μ*mol/L	279–320 *μ*mol/L	320–373 *μ*mol/L	>=373 *μ*mol/L		
Full–adjust^1^	1 ref	0.83 (0.66–1.05)	0.76 (0.60–0.96)	0.66 (0.51–0.84)	0.51 (0.39–0.67)	0.85 (0.77–0.93)	0.0004
Urate concentration in 2010	<246 *μ*mol/L	246–296 *μ*mol/L	296–342 *μ*mol/L	342–399 *μ*mol/L	>=399 *μ*mol/L		
Full‐adjust^1^	1 ref	0.53 (0.41–0.67)	0.64 (0.51–0.82)	0.45 (0.34–0.58)	0.33 (0.25–0.45)	0.65 (0.58–0.73)	<0.001
Change rate of urate during 2006–2010, *μ*mol/L per year	<−22	−22–0	0.1–17.1	17.2–38.5	>38.5		
Full‐adjust^1^	1 ref	0.69 (0.54–0.87)	0.58 (0.45–0.75)	0.50 (0.38–0.65)	0.53 (0.41–0.68)	0.87 (0.82–0.91)	<0.001

^1^ Adjusted for age, sex, educational level, income level, marital status, occupation, physical activity, smoking status, drinking status, tea consumption, myocardial infarction history, stroke history, cancer history, hypertension, diabetes, body mass index, triglyceride, low‐density lipoprotein, and high‐density lipoprotein.

^2^ Based on seven behavioral factors including sleep talking, shouting, limb movements, and sleep‐related injuries (score range 0–70, cutoff >7).

^3^ The cases who had pRBD occurring only during 2011–2012, the case number is 308.

**Figure 1:**
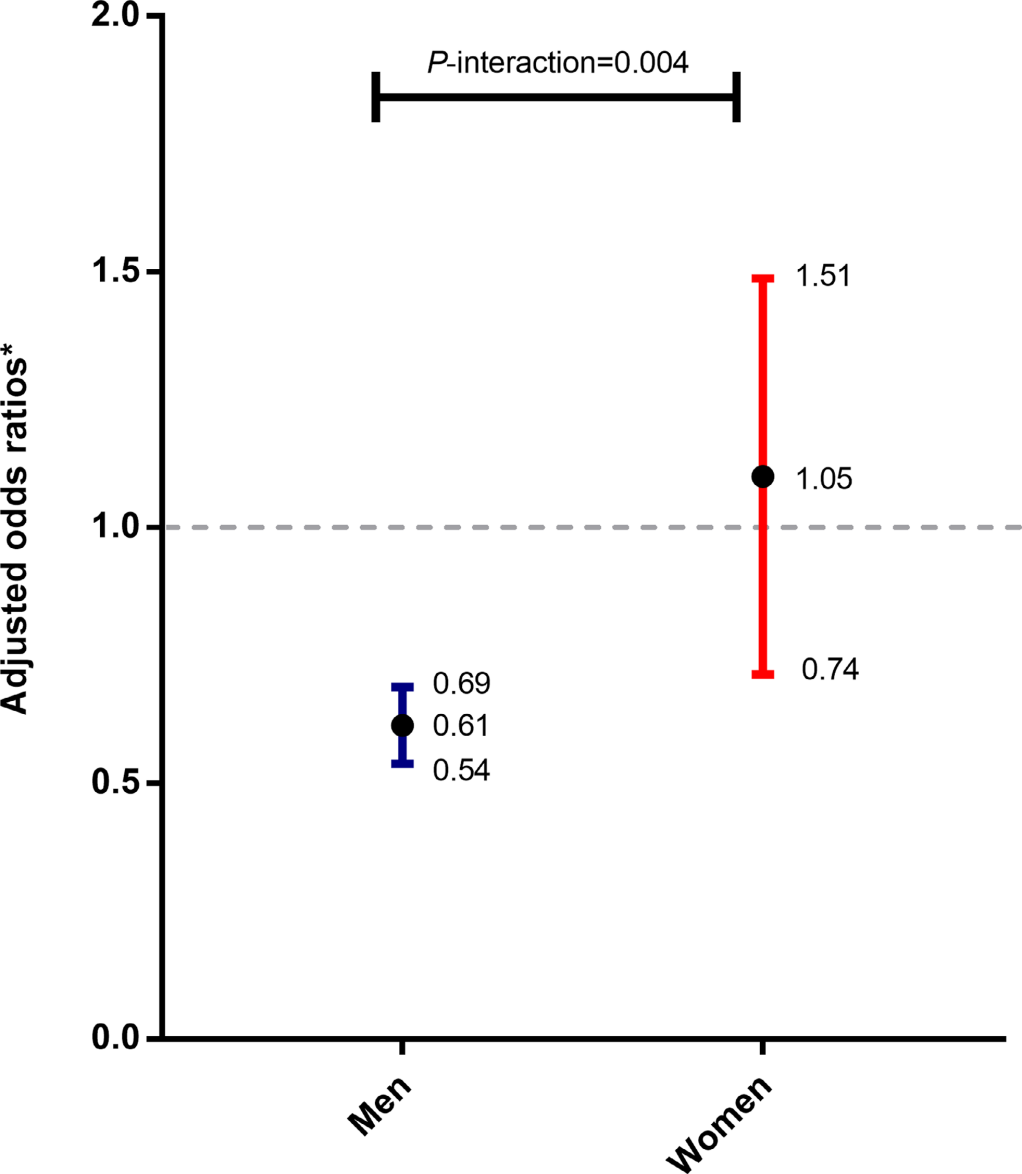
Adjusted odds ratios of having probable RBD of each 100 *μ*mol/L increase in urate. * adjusted for age, educational level, income level, marital status, occupation, physical activity, smoking status, drinking status, tea consumption, myocardial infarction history, stroke history, cancer history, hypertension, diabetes, body mass index, triglyceride, low‐density lipoprotein, and high‐density lipoprotein.

The observed association between urate concentration and pRBD risk appeared to be modified by sex (Fig. 1) – the adjusted OR values of each 100 *μ*mol/L increase in average urate between 2006 and 2010 for pRBD were 0.61(95% CIs 0.54–0.69) for men and 1.05(95% CIs 0.74–1.51) for women (*P*‐interaction = 0.004). In contrast, we did not observe significant interaction between urate and age, BMI, and hypertension in relation to pRBD (*P*‐interaction >0.2 for all).

## Discussion

In this community‐based study of 12,949 adults, we observed that individuals with higher urate concentrations had lower odds of having pRBD, compared to those with lower urate concentrations. The observed association appeared to be independent of potential risk factors for a vascular cause, such as BMI and hypertension, as well as other confounders, such as alcohol intake and occupation. This significant association between urate and pRBD was observed in men, but not in women. However, we did not observe significant association between urate concentrations in 2006–2010 and pRBD with onset after 2011.

Growing evidence has shown that RBD, reported aggregation of *α*‐synuclein,^21^, ^22^, ^23^ represents a unique opportunity to detect synucleinopathy pathology in its early stages.^24^ The International RBD Study Group found that the overall conversion rate from RBD to an overt neurodegenerative syndrome (parkinsonism as the first disease manifestation) was 6.3% per year, with 73.5% converting after 12‐year follow‐up.^3^ However, the natural history of prodromal RBD is not well known. Factors influencing a short or long latency between normal and onset of RBD are unknown.

The relation between urate and RBD was only examined in a recent study in which lower urate concentrations were observed in 42 individuals with idiopathic RBD (iRBD), relative to 45 controls (306 vs. 314 *μ*mol/L).^25^ However, the difference was not significant, possibly due to its small sample size (*P*‐difference = 0.17). An early study including 24 iRBD patients reported that higher urate concentrations were associated with longer RBD disease duration, without converting to PD.^26^

The biological mechanisms underlying the inverse relation between urate and RBD remain unclear. Previous investigations found that urate exhibited possible causal protective effects on PD. From a series of cohort studies, high urate concentrations were found to be associated with low risk of PD^6^, ^7^, ^8^, ^27^, ^28^, ^29^ or slower progression of PD.^8^, ^30^, ^31^, ^32^ These epidemiological data are consistent with laboratory studies showing that urate to be directly implicated in disease pathogenesis of synucleinopathies, and even more research suggested that urate could provide natural neuroprotection against the synucleinopathy. Urate within astrocytes prevented dopaminergic cell death and atrophy induced by oxidative and mitochondrial toxins in cellular PD models.^4^, ^33^ In vivo, urate may prevent death of dopaminergic cells in a mouse intrastriatal 6‐hydroxydopamine model of PD,^34^ and in a cellular model of the disease it attenuated rotenone toxicity.^35^ Of note, recent genetic studies generated inconsistent results regarding urate‐related genes and PD risk^36^, ^37^ and progression^32^ using the Mendelian randomization. However, these studies should be interpreted with caution due to a small effect of these genetic factors on urate status – explaining only ~5% of urate variance, which limits the power to detect the urate–PD relationship. Another experimental study found that urate administration was able to modulate the levels of autophagy markers, increase the autophagosome/autolysosome formation, and reduce *α*‐synuclein accumulation.^38^ The diminished levels of urate, in turn, could result in progressive accumulation of *α*‐synuclein, likely contributing to putative *α*‐synuclein‐related neurodegenerative processes (e.g., RBD and PD). Furthermore, recent imaging studies showed altered functional connectivity and neural network of motor activity in patients with RBD.^39^ Interestingly, urate level contributed to cortical functional networks in patients with early stage PD,^40^ which might also change in RBD.

There is also possibility that urate decline is in fact a consequence rather than the cause of the underlying neurodegenerative process. In our study, there was no significant association between average urate concentrations during 2006–2010 and pRBD onset after 2011. Urate can act like a prooxidant at higher concentrations or under other conditions. Low urate may rather be a marker of oxidative stress, which has been implicated as a core contributor to the initiation and progression of multiple neurodegenerative diseases.^41^ However, because of low prevalence of PD and relevant conditions in generate population, caution is needed in interpreting results. The Study of Urate Elevation in PD, Phase 3 (SURE‐PD3) was halted for futility, when no sign of urate's protective ability on PD progression was seen.^42^ However, the hypothesis needs to be investigated further in prodromal PD models (e.g., RBD) as a large proportion of dopaminergic neurons already died during onset of PD.

In our previous meta‐analysis, we found that men, but not women, with higher plasma urate concentration had a lower future risk of developing PD.^8^ Consistently, a similar sex difference for the urate–RBD relationship was observed in the current study. However, the exact underlying biological mechanisms for the observed sex difference remain unknown. It could be due to lower urate concentration in women, relative to men, or other determinants (e.g., diet and sexual hormones) for the sex difference in urate status. RBD is diagnosed much less commonly in women and only ~ 20% of the cohort participants are women. Lack of significant association in women could be thus due to lack of statistical power. The extra‐dopaminergic system could involve in the underlying biological basis – urate level was gender‐specifically associated with the resting‐state functional connectivity in de novo PD patients.^40^

Strengths of our study were a detailed demographic and clinical assessment in a large population. There are several limitations of the current study. We used a self‐report questionnaire to determine pRBD symptoms, which could not represent RBD and even misidentify. There were some false positive rates by the questionnaire‐based population studies. There were a large number of identified pRBD patients via the questionnaire who actually did not have RBD, as suggested by previous validation study.^14^, ^15^ Various sleep disorders, such as confusional arousals, sleep apnea, NREM parasomnias, and others, might mimic RBD symptoms. Higher urate concentration was associated with higher OSA risk,^43^ while lower urate concentration was associated with the presence and severity of chronic insomnia.^44^ However, excluding individuals with these sleep disorders did not materially change our results.

Generalizability is another limitation of our study because the current study is based on an industrial city in the North China. There is over one‐third coal miners in the cohort and the lifestyle differences are large. Some unmeasured confounds (smoking, diabetes, alcohol use, and genetic factors etc.) related to urate cannot be adjusted for and there is no consistent conclusion between them and RBD. In an earlier study, we reported an increased risk of pRBD in association with alcohol intake.^11^ Consistently, restricting to never‐drinkers in the current study did not change the observed association between urate and pRBD. As a cross‐sectional study, temporal relationships cannot be determined. However, we introduced some prospective components, by restricting to new‐onset pRBD. More diverse populations research is now needed to establish the association between RBD and urate and characteristics linked to urate.

Due to its observational study design, residual confounding is inevitable although we adjusted for a spectrum of potential confounders. We did not collect baseline data on using of antidepressant medications trigger symptoms of RBD. However, the frequency of antidepressant use in China is lower than in Western countries, even among individuals with a diagnosable mental illness.^45^ In 2016, we did the survey to update the general information on lifestyle factors and major chronic diseases, and for the first time collected information on physician‐diagnosed depression in the survey – only 0.13% of the Kailuan participants reported to have depression. Of note, compared to patients with "purely‐idiopathic" RBD, patients with antidepressant‐associated RBD had a lower risk of developing neurodegenerative disease.^46^ Another limitation is that incident PD cases were not documented in this cohort. We thus cannot study whether urate concentrations are associated with conversion to PD in participants with pRBD.

In conclusion, our study suggests that higher urate concentrations are associated with a lower likelihood of having pRBD, a well‐established prodromal PD symptom, which shares mechanistic features with PD. The result should be interpreted carefully because of possibility of residual confounding. One possible reason is that RBD or related pathological changes may generate low urate concentrations. Future studies with clinically confirmed RBD, and prospective study designs and longitudinal data would be appropriate to further investigate this association.

## Author Contributions

Xiang, Liufu, and Shouling contributed to the conception and design of the study; Junjuan and Songbin contributed to the acquisition and analysis of data; Yun, Michael, Milena, Alberto, and Xiang contributed to drafting the text and preparing the figures.

## Conflict of Interest

The authors report no disclosures relevant to the manuscript.

## Supporting information


**Table S1.** Basic characteristics in 2006 according to urate concentration.
