# Supplementary material for: Plasma urate concentrations and possible REM sleep behavior disorder
Source: Ann Clin Transl Neurol. 2019 Nov 12;6(12):2368–76. doi: 10.1002/acn3.50929 (PMC6917330; doi:10.1002/acn3.50929)
Supplement: Supplementary file 1 — Table S1. Basic characteristics in 2006 according to urate concentration. [file ACN3-6-2368-s001.docx]

|  | **Supplemental Table 1 Basic characteristics in 2006 according to urate concentration** | | | | | | | |
| --- | --- | --- | --- | --- | --- | --- | --- | --- |
| **Average urate concentration**  **Range, umol/L** | |  | **Q1**  **<243** | **Q2**  **243-290** | **Q3**  **291-334** | **Q4**  **335-397** | **Q5**  **≥388** | ***P* value** |
| **N** | | - | 2519 | 2882 | 2726 | 2632 | 2164 |  |
| **Men, No. (%)** | | - | 1644(65.26) | 2169(75.26) | 2357(86.46) | 2427(92.21) | 2083(96.26) | <0.001 |
| **Age, year*** | | - | 53.35±0.23 | 54.67±0.22 | 54.24±0.22 | 54.05±0.22 | 53.35±0.24 | 0.29 |
| **Educational level** | | Primary, No. (%) | 228(9.05) | 262(9.09) | 241(8.84) | 244(9.27) | 176(8.13) | 0.05 |
|  | | Middle, No. (%) | 2077(82.45) | 2376(82.44) | 2232(81.88) | 2117(80.43) | 1751(80.91) |  |
|  | | College, No. (%) | 214(8.50) | 244(8.47) | 253(9.28) | 271(10.30) | 237(10.95) |  |
| **Income level** | | <600/month, No. (%) | 797(31.91) | 895(31.37) | 939(34.73) | 926(35.51) | 752(34.85) | <0.001 |
|  | | 600-1000/month, No. (%) | 1498(59.97) | 1647(57.73) | 1446(53.48) | 1381(52.95) | 1153(53.43) |  |
|  | | >1000/month, No. (%) | 203(8.13) | 311(10.90) | 319(11.80) | 301(11.54) | 253(11.72) |  |
| **Marital status** | | Single, No. (%) | 42(1.68) | 36(1.26) | 45(1.66) | 44(1.69) | 54(2.50) | 0.14 |
|  | | Married, No. (%) | 2386(95.55) | 2741(95.97) | 2589(95.68) | 2501(95.90) | 2045(94.76) |  |
|  | | Divorced, No. (%) | 69(2.76) | 79(2.77) | 72(2.66) | 63(2.42) | 59(2.73) |  |
| **Occupation** | | White collar, No. (%) | 191(7.65) | 192(6.73) | 200(7.39) | 175(6.72) | 178(8.26) | <0.001 |
|  | | Blue collar, No. (%) | 1537(61.53) | 1592(55.78) | 1290(47.69) | 1164(44.70) | 1008(46.77) |  |
|  | | Coalminer, No. (%) | 770(30.82) | 1070(37.49) | 1215(44.92) | 1265(48.58) | 969(44.97) |  |
| **Physical activity**  **(Every time more than 20 min)** | | Never, No. (%) | 266(10.65) | 248(8.70) | 233(8.62) | 201(7.71) | 202(9.37) | <0.001 |
|  |  | <4 times/week, No. (%) | 1960(78.49) | 2224(78.01) | 2051(75.85) | 1988(76.26) | 1640(76.07) |  |
|  |  | 4+ times/week, No. (%) | 271(10.85) | 379(13.29) | 420(15.53) | 418(16.03) | 314(14.56) |  |
| **Smoking status** | | Never, No. (%) | 1723(68.40) | 1601(55.55) | 1179(43.25) | 995(37.80) | 702(32.44) | <0.001 |
|  | | Past smoker, No. (%) | 92(3.65) | 159(5.52) | 199(7.30) | 220(8.36) | 222(10.26) |  |
|  | | Current smoker, No. (%) | 704(27.95) | 1122(38.93) | 1348(49.45) | 1417(53.84) | 1240(57.30) |  |
| **Drinking status** | | Never, No. (%) | 1650(65.50) | 1551(53.82) | 1098(40.28) | 836(31.76) | 527(24.35) | <0.001 |
|  | | Past drinker, No. (%) | 76(3.02) | 105(3.64) | 134(4.92) | 129(4.90) | 105(4.85) |  |
|  | | Current drinker, No. (%) | 793(31.48) | 1226(42.54) | 1494(54.81) | 1667(63.34) | 1532(70.79) |  |
| **Tea consumption** | | Never, No. (%) | 1892(75.11) | 2056(71.34) | 1767(64.82) | 1606(61.02) | 1193(55.13) | <0.001 |
|  | | <4 times/week, No. (%) | 431(17.11) | 528(18.32) | 604(22.16) | 625(23.75) | 571(26.39) |  |
|  | | 4+ times/week, No. (%) | 196(7.78) | 298(10.34) | 355(13.02) | 401(15.24) | 400(18.48) |  |
| **MI history** | | Yes, No. (%) | 18(0.71) | 24(0.83) | 28(1.03) | 29(1.10) | 46(2.13) | <0.001 |
| **Stroke history** | | Yes, No. (%) | 45(1.79) | 69(2.39) | 79(2.90) | 83(3.15) | 86(3.97) | <0.001 |
| **Cancer history** | | Yes, No. (%) | 11(0.44) | 10(0.35) | 8(0.29) | 7(0.27) | 7(0.32) | 0.86 |
| **Hypertension** | | NO, No. (%) | 795(31.56) | 918(31.85) | 874(32.06) | 696(26.44) | 519(23.98) | <0.001 |
|  | | Prehypertension, No. (%) | 927(36.80) | 1057(36.68) | 1061(38.92) | 1044(39.67) | 814(37.62) |  |
|  | | Yes, No. (%) | 797(31.64) | 907(31.47) | 791(29.02) | 892(33.89) | 831(38.40) |  |
| **Diabetes** | | NO, No. (%) | 1819(72.21) | 2132(73.98) | 2090(76.67) | 1979(75.19) | 1688(78.00) | <0.001 |
|  | | Prediabetes, No. (%) | 430(17.07) | 478(16.59) | 437(16.03) | 467(17.74) | 361(16.68) |  |
|  | | Yes, No. (%) | 270(10.72) | 272(9.44) | 199(7.30) | 186(7.07) | 115(5.31) |  |
| **head injury** | | Yes, No. (%) | 74(2.94) | 88(3.05) | 68(2.49) | 66(2.51) | 37(1.71) | 0.03 |
| **Body Mass Index (BMI)*, kg/m^2^** | |  | 24.13±0.07 | 24.55±0.06 | 24.96±0.06 | 25.71±0.07 | 26.50±0.07 | <0.001 |
| **Triglyceride*, mmol/L** | |  | 1.42±0.03 | 1.46±0.02 | 1.47±0.02 | 1.66±0.02 | 2.00±0.03 | <0.001 |
| **LDL_C*, mmol/L** | |  | 2.24±0.01 | 2.21±0.01 | 2.20±0.01 | 2.23±0.01 | 2.24±0.02 | 0.17 |
| **HDL_C*, mmol/L** | |  | 1.60±0.01 | 1.58±0.01 | 1.56±0.01 | 1.54±0.01 | 1.53±0.01 | <0.001 |
|  | Note: LDL_C, low density lipoprotein; HDL_C, high-density lipoprotein.  * Mean±stand error, adjusted for age and sex | | | | | | | |
